# Natural metabolites used in traditional Chinese medicine for cardiovascular diseases: pharmacological mechanisms, evidence, and future directions

**DOI:** 10.3389/fphar.2025.1656751

**Published:** 2025-11-10

**Authors:** Xinyu Liu, Wenfeng Zhang, Xiao Miao, Yinghua Hu

**Affiliations:** 1 School of Basic Medical Sciences, Changchun University of Chinese Medicine, Changchun, Jilin, China; 2 College of Acupuncture and Tuina, Changchun University of Chinese Medicine, Changchun, Jilin, China; 3 Department of Ophthalmology, The Second Hospital of Jilin University, Jilin, China

**Keywords:** Chinese medicine, natural metabolites, CVDs, flavonoids, alkaloids, saponins

## Abstract

Cardiovascular diseases (CVDs) remain the leading cause of death and disability worldwide, highlighting an urgent need for new treatments. Traditional Chinese Medicine (TCM) offers a rich repository of natural metabolites (flavonoids, alkaloids, saponins, etc.) that act on multiple targets to protect the heart and blood vessels. These compounds have demonstrated multiple cardioprotective effects, including anti-inflammatory, antioxidant, anti-atherosclerotic, and blood pressure–lowering activities. They work by reducing oxidative stress, dampening chronic inflammation, improving blood vessel function, correcting abnormal lipid levels, and mitigating cardiac fibrosis. Recent preclinical studies and clinical trials show that TCM-derived metabolites can improve cardiovascular health. For instance, the multi-herb formula Qili Qiangxin and the alkaloid berberine have improved heart failure symptoms and cardiac function in clinical trials when added to standard therapy. These examples underscore the clinical potential of TCM compounds. However, challenges like poor bioavailability, complex multi-component interactions, and lack of standardization still hinder their widespread use. To address these issues, researchers are exploring advanced drug delivery methods and better quality control with modern analytical tools. If these hurdles are overcome, TCM-derived therapies could be successfully integrated into mainstream cardiovascular care, offering a novel multi-target approach to combat CVDs.

## 1 Introduction

Cardiovascular diseases (CVDs) remain the leading cause of mortality and disability worldwide, imposing an enormous health and economic burden. Globally, CVDs account for roughly one-third of all deaths (approximately 17–18 million per year). The rising prevalence of hypertension, diabetes, obesity, and other risk factors has led to tens of millions of new CVDs cases annually. Low- and middle-income countries are disproportionately affected due to limited healthcare access and resource constraints, but high-income regions also face growing challenges as populations age and lifestyle-related risk factors persist. Despite advances in prevention and acute care, the overall CVDs burden continues to increase, underscoring the urgent need for more effective and comprehensive strategies to combat these diseases (World Health Organization, 2021; Khan et al., 2024; Centers for Disease Control and Prevention, 2024; Martin Ss Fau - Aday et al., 2025).

Conventional Western therapies for CVDs—such as antihypertensive drugs, statins for dyslipidemia, antiplatelet agents, and neurohormonal blockers for heart failure—have significantly improved patient outcomes. However, these standard treatments have important limitations and unmet clinical needs. Many drugs have a single mechanism of action and are limited in their effects on complex pathological changes. Patients often experience side effects (for example, persistent cough with ACE inhibitors or muscle pain with high-dose statins), issues with adherence, and even drug resistance or tolerance over time. Furthermore, residual risk remains high; even with optimal medical therapy, substantial proportions of patients continue to suffer events such as myocardial infarction or progression of heart failure. These challenges highlight the need for complementary or alternative therapeutic approaches that can safely target additional pathological processes (e.g., inflammation, fibrosis) beyond the reach of current standard-of-care medications.

Many foods have the function of promoting health, so the concept of “dietotherapy” or “medicinal food” is widely known, and this phenomenon is called “continuum of food and medicine” by academic circles; Together with traditional Chinese medicine (TCM) and its natural drug metabolites as potential resources for new CVD therapy, it is attracting increasing academic attention (Chen, 2023; Yao et al., 2023). TCM-derived natural products—isolated from botanical drugs (medicinal plants) long used in TCM practice—offer multi-target and multi-pathway mechanisms that could holistically address the complex pathophysiology of CVDs. Many such metabolites (e.g., flavonoids from *Scutellaria baicalensis* and citrus peels, saponins from *Panax ginseng*, *Astragalus membranaceus* and Dioscorea nipponica Makino, alkaloids like berberine from *Coptis chinensis*) have demonstrated cardioprotective effects with relatively favorable safety profiles. These phytochemicals can concurrently modulate oxidative stress, inflammation, endothelial function, lipid metabolism, and myocardial remodeling, thereby intervening at multiple disease nodes (Tang et al., 2015a; Fan et al., 2016; Feng et al., 2017; Ou-Yang et al., 2018; Zeng et al., 2019; Orgah et al., 2020; Shao et al., 2020; Sadoughi et al., 2021; Jin et al., 2024; Shi et al., 2024). TCM treatments involve multiple components and targets acting concurrently, making their mechanisms difficult to fully elucidate. Furthermore, comprehensive reviews focusing on the active compounds from TCM and their cardiovascular mechanisms remain limited.

**FIGURE 1:**
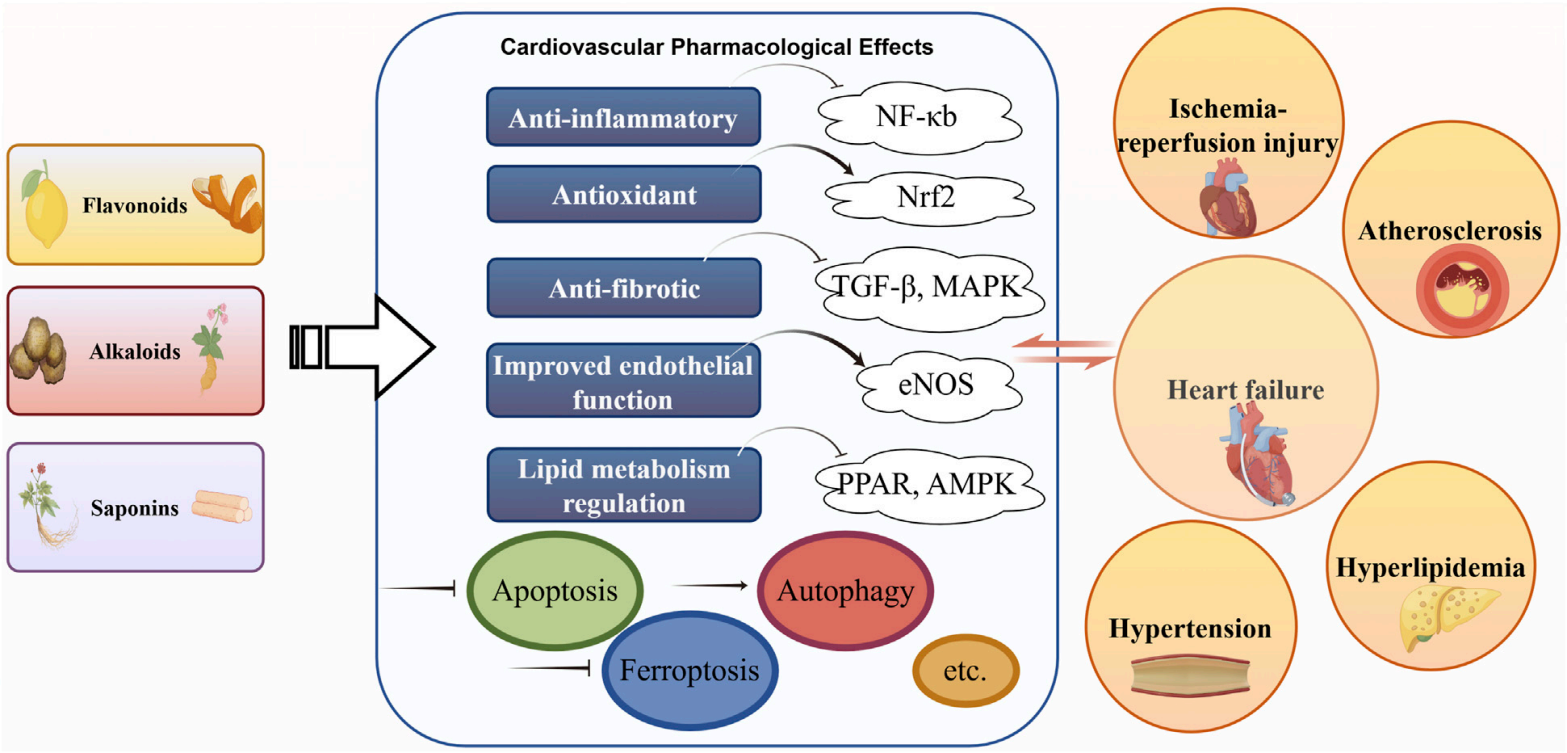
Main classes of TCM natural compounds and cardiovascular mechanisms. Explanation: Schematic diagram highlighting three primary classes of Traditional Chinese Medicine (TCM)–derived natural compounds and their cardioprotective mechanisms and disease targets. The figure illustrates how each class exerts beneficial effects on the cardiovascular system. Abbreviations in the figure are defined as follows: TCM (Traditional Chinese Medicine); CVD (cardiovascular disease); MI (myocardial infarction); I/R (ischemia–reperfusion) (Tang et al., 2015a; Feng et al., 2017; Ou-Yang et al., 2018; Shao et al., 2020).

This review explores the application of natural metabolites used in TCM in the prevention and treatment of major cardiovascular diseases, highlighting their pharmacological mechanisms and therapeutic potential. We first provide a brief background on the multi-target cardiovascular effects of TCM-derived metabolites (Figure 1), then discuss evidence for their benefits in specific disease contexts (atherosclerosis, hypertension, myocardial infarction, heart failure), integrate findings from clinical studies, and finally consider the challenges and future directions for translating these therapies into mainstream cardiovascular care.

A systematic literature search was performed across the PubMed database for relevant studies published up to June 2025. The search employed a combination of MeSH terms and free text keywords, including “cardiovascular disease” and the names of specific botanical agents and their bioactive constituents (e.g., “berberine,” “ginseng,” “flavonoid”). Inclusion criteria were restricted to peer-reviewed articles published in English within the preceding 5 years. Studies encompassing *in vitro* assays, *in vivo* animal models utilizing traditional Chinese medicine (TCM), and clinical trials investigating TCM for cardiovascular diseases [CVDs] were eligible for inclusion. To ensure taxonomic accuracy, all botanical identities were cross-referenced with the Kew Medicinal Plant Names Service. Furthermore, the reporting of plant materials and extracts conformed to the ConPhyMP guidelines. Supplementary Tables S1, S2 provide a synopsis of the included botanical drugs, their preparation methods, and analytical characterization. A summary of key TCM-derived metabolites, along with their sources and molecular targets, is presented in Tables 1–3. Table 4 collates key clinical studies meeting the inclusion criteria. Subsequently, the discussion is organized according to major cardiovascular conditions, featuring representative metabolites from prominent chemical classes (i.e., flavonoids, alkaloids, and saponins) that have demonstrated cardioprotective effects in relevant experimental models.

**TABLE 1:** Representative Flavonoids and their main sources and pharmacological effects of traditional Chinese medicine.

Chinese name	Latin name	Main flavonoids	Pharmacological effects
Ban Lan Gen	*Isatis tinctoria*	Luteoli, Quercetin	Anti-inflammatory, antiviral, vascular protection
Chen Pi	*Citrus reticulata*	Hesperetin, Naringenin	Lowering blood lipids, anti-atherosclerosis
Gan Cao	*Glycyrrhiza uralensis*	Licochalcone A, Isoliquiritigenin	Antioxidant, anti-inflammatory, immunomodulatory
Ge Gen	*Pueraria lobata*	Puerarin	Dilate blood vessels, improve microcirculation, and lower blood pressure
Hu Pi	*Quercus spp.*	Quercetin	Antioxidant, antiplatelet aggregation, lipid-lowering
Hu Zhang	*Polygonum cuspidatum*	Quercetin	Antioxidant, antiplatelet aggregation, anti-atherosclerosis
Huang Jing	*Polygonatum sibiricum*	Rutin, Isoquercitrin	Immunomodulation, anti-inflammatory, hypoglycemic
Huai Hua	*Sophora japonica*	Rutin, Quercetin	Lowering blood pressure, anticoagulation, anti-oxidation
Huang Hua Hao	*Artemisia annua*	Naringenin, Luteolin	Anti-inflammatory, immunomodulatory, anti-vascular sclerosis
Huang Qin	*Scutellaria baicalensis*	Baicalin, Baicalein	Anti-oxidation, anti-inflammatory, improve vascular function
Jue Ming Zi	*Cassia obtusifolia*	Rutin	Antioxidant, lipid-lowering, anti-inflammatory
Ku Qiao	*Fagopyrum tataricum*	Rutin	Lowering blood lipids, anti-diabetes, protecting vascular endothelium
Sang Ye	*Morus alba*	Isoquercitrin	Lower blood sugar, anti-oxidation, improve microcirculation
Wu Wei Zi	*Schisandra chinensis*	Naringenin	Regulate cardiovascular function, anti-oxidation, protect myocardium
Yin Xing Ye	*Ginkgo biloba*	Ginkgoflavones	Protect blood vessels, prevent platelet aggregation, and prevent stroke
Zi Su	*Perilla frutescens*	Perilloflavone, Quercetin	Anti-inflammatory, anti-allergic, lipid-lowering

**TABLE 2:** Representative alkaloids and their main sources and pharmacological effects of traditional Chinese medicine.

Chinese name	Latin name	Major alkaloids	Pharmacological effects
Ban Xia	*Pinellia ternata*	Pinelline	Antiemetic and expectorant, but toxic in raw form, requires processing before use
Bei Mu	*Fritillaria thunbergii*	Peimine	Expectorant, anti-inflammatory
Chuan Wu	*Aconitum carmichaelii*	Aconitine	Strong analgesic, but highly toxic, requires proper processing before use
Huang Lian	*Coptis chinensis*	Berberine	Antibacterial, anti-inflammatory, lipid-lowering, hypoglycemic, gut microbiota regulation, cardiovascular protection, antioxidant
Gou Teng	*Uncaria rhynchophylla*	Rhynchophylline	Antihypertensive, sedative, neuroprotective
Ku Mu	*Picrasma quassioides*	Quassin	Heat-clearing, detoxifying, antidiarrheal
Ku Shen	*Sophora flavescens*	Matrine, Oxymatrine	Anti-inflammatory, antiviral, antitumor, hepatoprotective
Ma Huang	*Ephedra sinica*	Ephedrine	Bronchodilator, diuretic, hypertensive
Ma Qian Zi	*Strychnos nux-vomica*	Strychnine	Central nervous system stimulant, used for neurological disorders, but highly toxic
Man Tuo Luo	*Datura metel*	Scopolamine, Hyoscyamine	Antispasmodic and analgesic effects, but toxic, should be used under medical supervision
Nan Tian Zhu	*Nandina domestica*	Nantenine	Heat-clearing, detoxifying, antitussive
Shi Song	*Lycopodium japonicum*	Lycopodine, Huperzine A	Neuroprotective, anti-inflammatory, analgesic
Wu Tou	*Aconitum carmichaelii*	Acnitine	Anti-rheumatic, analgesic
Yan Hu Suo	*Corydalis yanhusuo*	Dehydrocorybulbine	Analgesic, blood circulation improvement
Yan Ling Cao	*Trillium*	Colchicine	Anti-inflammatory, cardiovascular protection, antioxidant
Yi Mu Cao	*Leonurus japonicus*	Leonurine	Blood circulation improvement, antihypertensive, cardioprotective

**TABLE 3:** Representative Saponins and their main sources and pharmacological effects of traditional Chinese medicine.

Chinese name	Latin name	Major alkaloids	Pharmacological effects
Dan Shen	*Salvia miltiorrhiza*	Danshen saponins	Widely used for cardiovascular diseases; enhances microcirculation, dilates coronary arteries, reduces blood lipid levels, and prevents atherosclerosis
Di Huang	*Rehmannia glutinosa*	Catalpol, Rehmanniosides	Impacts heart health by reducing blood pressure and cholesterol, also shown to improve cardiac function in diabetic patients
He Shou Wu	*Polygonum multiflorum*	Tuberfleeceflower saponins	Influences lipid metabolism and has been linked to cardiovascular health by potentially reducing the risk of arteriosclerosis
Huang Qi	*Astragalus membranaceus*	Astragaloside IV	Protects myocardial cells, reduces hypertension, and acts as a diuretic to treat chronic heart failure
Jie Geng	*Platycodon grandiflorus*	Platycodon saponins	Helps to reduce cholesterol levels and improves lung health, which indirectly supports heart function
Mai Dong	*Ophiopogon japonicus*	Ophiopogonin	Primarily used for its cardiovascular benefits such as enhancing coronary blood flow and protecting against ischemic injuries
Ren Shen	*Panax ginseng*	Ginsenosides	Enhances cardiovascular performance by modulating blood pressure, improving blood circulation, and reducing myocardial oxygen consumption
San Qi	*Panax notoginseng*	Notoginsenosides	Promotes blood circulation, reduces blood clot formation, and is used for treating coronary artery diseases and ischemic strokes
Shan Zhu Yu	*Cornus officinalis*	Cornus saponins	Known for anti-inflammatory and antioxidant effects that benefit heart health, particularly in ischemic conditions
Shan Yao	*Dioscorea opposita*	Diosgenin	Known for its benefits in regulating cholesterol levels, enhancing endocrine function, and its potential in improving cardiovascular health through anti-inflammatory and antioxidant properties
Yu Zhu	*Polygonatum odoratum*	Polygonatum saponins	Used for its cardiovascular protective effects, reduces heart rate, and has been shown to decrease blood pressure

**TABLE 4:** Comparison of active compound categories in traditional Chinese medicine.

Category	Key pharmacological effects and mechanisms	Pharmacological characteristics
Flavonoids	Antioxidant: Activates the Nrf2/ARE pathway, upregulating antioxidant enzymes (SOD, GPx) to reduce oxidative stress. Anti-inflammatory: Inhibits NF-κB signaling, reducing IL-6 and TNF-α expression. Endothelial Function Improvement: Enhances eNOS/NO signaling, promoting nitric oxide synthesis for vasodilation. Lipid Metabolism Regulation: Modulates PPAR-γ expression, reducing LDL oxidation and cholesterol levels. Antiplatelet Activity: Inhibits COX-1/COX-2 pathways to reduce platelet aggregation	Acts on multiple pathways simultaneously, moderate bioavailability, often requiring modifications to improve solubility and absorption
Alkaloids	Neuropharmacological Effects: Interacts with cholinergic, adrenergic, and opioid receptors, affecting neurotransmission. Cardiovascular Regulation: Blocks calcium channels and regulates β-adrenergic receptors, reducing hypertension and arrhythmias. Anti-inflammatory: Inhibits Toll-like receptor 4 (TLR4) and NF-κB pathways, reducing inflammatory cytokines. Antibacterial and Antiviral: Targets bacterial DNA gyrase and viral replication enzymes, inhibiting pathogen growth	Lipophilic, easily crosses the blood-brain barrier, significant central nervous system effects, some compounds require toxicity assessment
Saponins	Cardioprotective: Enhances eNOS/NO signaling, promoting vasodilation and reducing hypertension. Anti-inflammatory: Inhibits NF-κB and MAPK pathways, reducing cytokine production (TNF-α, IL-6). Lipid Metabolism Regulation: Modulates PPAR-γ and NPC1L1 pathways, improving cholesterol homeostasis. Anti-fibrotic: Suppresses TGF-β/Smad signaling, preventing myocardial and vascular fibrosis. Immunomodulatory: Regulates TLR4/NF-κB, balancing immune responses	Multi-target action with broad physiological effects, poor bioavailability due to extensive metabolism, modifications such as nanoparticle delivery improve absorption and efficacy

## 2 Preclinical evidence and mechanistic studies

This section summarizes the key *in vitro* and *in vivo* findings from preclinical studies of major classes of natural metabolites from TCMs with cardioprotective effects, and discusses the key molecular targets and pathways by which these natural metabolites exert their cardioprotective effects. Notably, these natural compounds often act through multiple pathways in parallel, rather than through a single target. Common mechanisms include enhancing antioxidant defenses (typically through activation of the Nrf2/ARE pathway), inhibiting pro-inflammatory signaling (e.g., NF-κB and MAPK pathways), increasing nitric oxide (NO) bioavailability in endothelial cells, regulating lipid metabolism, and alleviating pathological fibrosis.

### 2.1 Multi-target cardiovascular benefits of TCM natural compounds

Natural metabolites from TCM exhibit multifaceted cardioprotective properties that set them apart from single-target conventional drugs. These bioactive molecules often act on numerous molecular pathways simultaneously, conferring broad therapeutic effects across the cardiovascular system. A unifying feature is their ability to mitigate oxidative stress—for example, many flavonoids, alkaloids, and saponins activate the Nrf2/ARE pathway, upregulating endogenous antioxidant enzymes (such as superoxide dismutase and glutathione peroxidase) to neutralize excess reactive oxygen species (Wang Q. W. et al., 2019; Liu et al., 2021; Oluranti et al., 2021; Ren et al., 2021; Shi et al., 2021; Tan et al., 2021; Obeidat et al., 2022; Qi et al., 2022; Wang S. H. et al., 2022; Wei et al., 2022; Fang et al., 2024; Feng et al., 2024; Guo et al., 2024; Liu Z. et al., 2024; Sun et al., 2024; Yao et al., 2024). Through antioxidant action, these metabolites preserve endothelial function and prevent oxidative damage to vessel walls and myocardium. In parallel, Natural metabolites such as flavonoids (e.g. quercetin, luteolin) and alkaloids (e.g. berberine) often exert anti-inflammatory effects by inhibiting pro-inflammatory signaling pathways like NF-κB, MAPK, and PI3K/Akt (Shen S. et al., 2023; Chen S. et al., 2024; Tang J. et al., 2024; Tuo et al., 2024). This results in lower expression of cytokines (TNF-α, IL-6, etc.) and adhesion molecules, dampening the vascular inflammation that drives atherosclerosis and hypertensive organ damage (Wang Q. et al., 2020; Muvhulawa et al., 2022; Shao et al., 2022; Barreca et al., 2023; Hassanein et al., 2023; Sun et al., 2023; Yang L. et al., 2023; Fang et al., 2024; Tuo et al., 2024; Wang A. et al., 2024). Many TCM metabolites also improve endothelial function and vasodilation: for instance, flavonoids and saponins promote nitric oxide (NO) production via endothelial NO synthase (eNOS) activation, improving NO bioavailability (Grassi et al., 2013; Martínez-Fernández et al., 2015; Bondonno et al., 2016; Heidary Moghaddam et al., 2020; Lin et al., 2020; Jalili et al., 2024). The resulting vasorelaxation translates into improved blood flow and reduced blood pressure (Adetunji et al., 2023; Li and Zhang, 2023; Harahap et al., 2024; Liu Y. et al., 2024; Miao et al., 2024; Tang F. et al., 2024; Wang Z. et al., 2024). Additionally, several natural metabolites beneficially modulate lipid metabolism – they can inhibit cholesterol absorption, upregulate LDL receptors, and improve lipid profiles by lowering LDL and triglycerides while raising HDL (Assini et al., 2013; Kianbakht et al., 2014; Kuipers et al., 2018; Hou et al., 2020; Wang Q. et al., 2024). By correcting dyslipidemia, they help curb atherosclerotic plaque formation (Panchal et al., 2011; Kwok et al., 2022; Wani et al., 2023; Lv et al., 2024; Mu et al., 2024; Wan et al., 2024; Zhao et al., 2024). Finally, many exhibit anti-fibrotic and cardiomyocyte-protective actions: they interfere with fibrosis-related pathways (TGF-β/Smad, etc.), inhibit cardiac fibroblast activation, and reduce pathological deposition of extracellular matrix in the heart (Xiao et al., 2018; Li et al., 2021; Jia et al., 2022; Wang M. et al., 2023; Lin et al., 2024). Some even promote adaptive autophagy or have positive inotropic effects that support cardiac function (Wang L. et al., 2019; Wang et al., 2021; Qu et al., 2022; Yao et al., 2022; Abudureyimu et al., 2023; Shen F. et al., 2023; Zhan et al., 2023; Erfu et al., 2024). Collectively, the ability of TCM-derived metabolites to address multiple pathogenic factors of CVD concurrently (as summarized in Figure 1) holds promise for treating complex cardiovascular disorders where no single molecular target is sufficient (Zhang et al., 2024). A comprehensive intervention strategy is particularly well-suited for addressing the intricate pathogenesis of complex cardiovascular disease, where targeting a solitary molecule proves inadequate. Within this context, it is noteworthy that various natural compounds interact with essential metabolic nuclear receptors, among them the peroxisome proliferator-activated receptors (PPARs). Specifically, diverse alkaloids and saponins have been shown to activate both PPARα and PPARγ isoforms. Upon activation, these receptors exert dual functionality by inhibiting NF-κB-driven inflammatory cascades and promoting favorable alterations in lipid metabolism. As such, the engagement of PPAR pathways constitutes a critical mechanism underlying the multi-target cardioprotective properties of TCM metabolites.

### 2.2 Atherosclerosis and coronary artery disease

Atherosclerosis is a chronic arterial disease characterized by the buildup of lipid-rich plaques in the vessel wall, leading to coronary artery disease and other ischemic complications. Endothelial injury and dysfunction initiate the process, permitting low-density lipoprotein (LDL) particles to infiltrate the intima and become oxidized. Oxidized LDL triggers an inflammatory cascade – monocytes migrate into the intima and differentiate into macrophages, engulfing lipids to form foam cells. A fatty streak evolves as these foam cells accumulate, and ongoing inflammation drives the expansion and instability of plaques. Key contributors to atherogenesis include oxidative stress (excess ROS oxidizing lipids and damaging endothelial cells), vascular inflammation (cytokine and chemokine release recruiting more immune cells), endothelial dysfunction (reduced NO bioavailability and loss of barrier integrity), and dyslipidemia (elevated LDL/triglycerides and low HDL). Over time, plaques can narrow arteries or rupture, causing thrombosis and acute myocardial infarction (Ajoolabady et al., 2024).

Natural products used in TCM have shown significant anti-atherosclerotic activities in preclinical studies by simultaneously counteracting the aforementioned pathogenic mechanisms (Si and Lai, 2024). A prominent example is the class of flavonoids, which includes metabolites from *Scutellaria baicalensis* (Huang Qin; e.g., baicalin, baicalein), *Pueraria lobata* (Ge Gen; puerarin), *Ginkgo biloba* (Yin Xing Ye; quercetin, ginkgoflavones), *Morus alba* (Sang Ye; isoquercitrin, morusin), *Citrus reticulata* (Chen Pi; hesperetin, naringenin), *Perilla frutescens* (Zi Su Ye; perilloflavone, quercetin), *Glycyrrhiza uralensis* (Gan Cao; licochalcone A, isoliquiritigenin), and *Sophora japonica* (Huai Hua; rutin, quercetin), etc. Flavonoids prevent the progression of atherosclerosis by scavenging free radicals and activating antioxidant defenses (Nrf2-mediated), thereby reducing LDL oxidation and endothelial damage. They also inhibit the inflammatory component of plaque formation; *in vitro* studies demonstrate that flavonoids suppress NF-κB signaling and lower pro-inflammatory mediator levels in vascular cells, which in turn mitigates leukocyte adhesion and foam-cell formation (Zhang et al., 2021; Ding et al., 2024; Wang Y. M. et al., 2024). In addition, flavonoid-rich botanical drugs have been noted to improve lipid profiles. For instance, puerarin (from *P. lobata*) and rutin (from *S. japonica*) can upregulate hepatic LDL receptors and promote cholesterol clearance, leading to reduced circulating LDL levels. In some animal studies, increased flavonoid intake has been inversely associated with the development of atherosclerosis (Bondonno et al., 2024).

Saponins (glycosidic metabolites from TCM botanical drugs like *Panax notoginseng*) likewise display broad anti-atherosclerotic effects. *Panax notoginseng* saponins (PNS), for example, have been shown in rats and rabbits to reduce atherosclerotic plaque development via multiple pathways. PNS exert antioxidant and anti-inflammatory actions similar to flavonoids, and notably they inhibit endothelial activation: in one endothelial cell study, notoginsenoside R1 and ginsenoside Rb1 from *Panax* blocked p38 MAPK signaling and downregulated vascular cell adhesion molecule-1 (VCAM-1) expression, thus preventing monocyte adhesion and plaque initiation (Fan et al., 2016). PNS have also been reported to inhibit foam-cell formation and ferroptosis within plaques, stabilizing the lesions. These combined effects led to markedly attenuated plaque progression in PNS-treated animals (Zhao et al., 2024).

**FIGURE 2:**
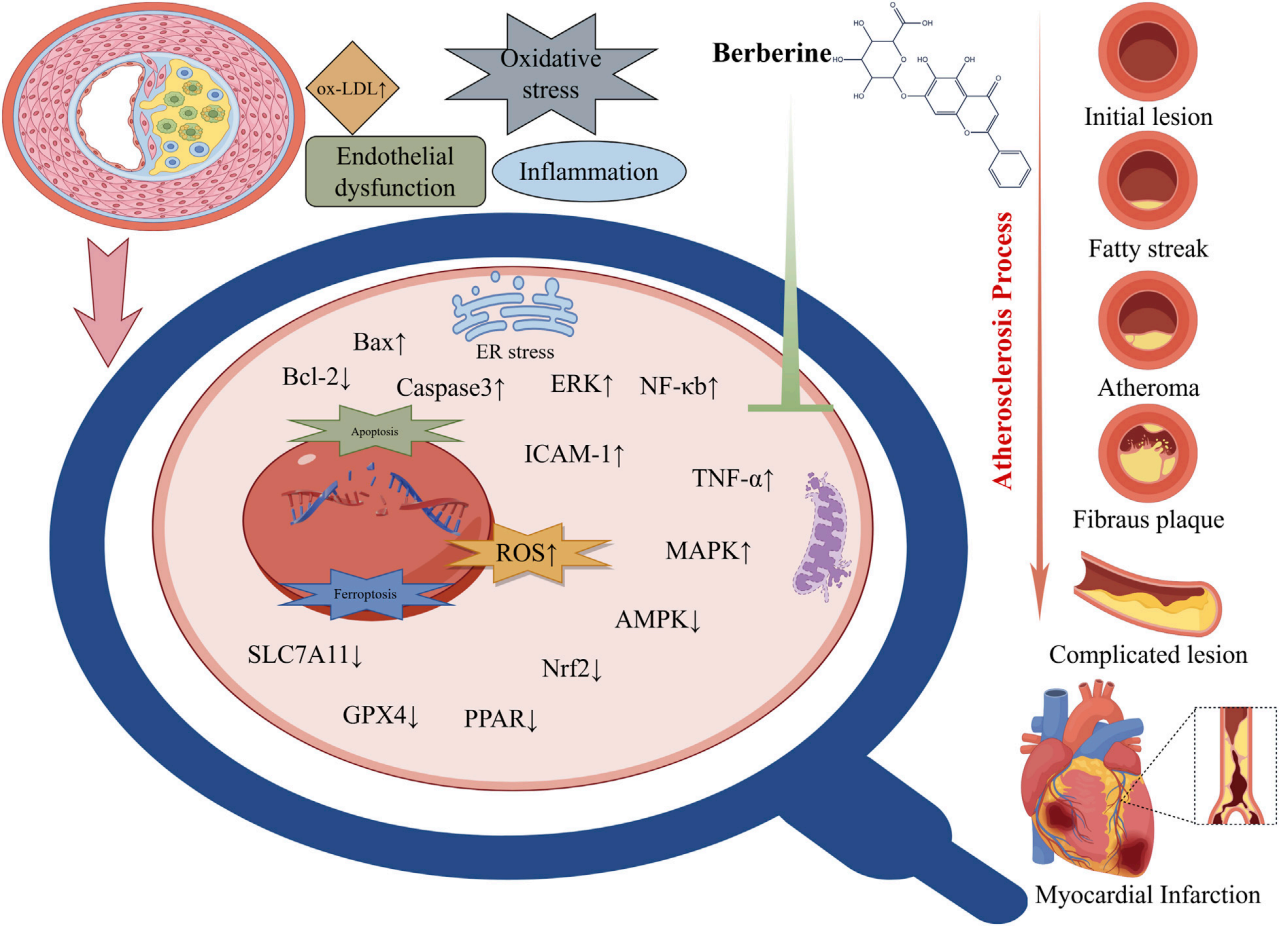
Molecular targets and cardioprotective mechanisms of berberine. Explanation: Key molecular actions of berberine include activation of AMPK (AMP-activated protein kinase) and related signaling pathways, which enhances endothelial nitric oxide synthase (eNOS) activity and nitric oxide (NO) bioavailability, leading to improved endothelial function and vasodilation. Berberine also inhibits pro-inflammatory signaling cascades such as NF-κB and MAPK, thereby reducing the production of inflammatory cytokines and oxidative stress in blood vessels. These anti-inflammatory and antioxidant effects help prevent endothelial damage and plaque formation, conferring protection against atherosclerosis and ischemic injury. In cardiac muscle, berberine attenuates cardiomyocyte apoptosis and enhances myocardial energy metabolism, partly through the AMPK–Akt–eNOS and other cardioprotective pathways. Through these combined actions – improving endothelial function, lowering blood lipids, suppressing inflammation, and stabilizing cardiac electrophysiology – berberine provides broad cardioprotection (Abbreviations: BBR, berberine; AMPK, AMP-activated protein kinase; eNOS, endothelial nitric oxide synthase; NF-κB, nuclear factor kappa-B; NO, nitric oxide; MAPK, mitogen-activated protein kinase; all other abbreviations are defined in the text or figure legend).

Similarly, alkaloids from TCM offer anti-atherogenic benefits. Colchicine is a small-molecule alkaloid drug that has been used in clinical atherosclerosis treatment (as an anti-inflammatory). It is derived from plants such as *Colchicum* and *Trillium* and has anti-inflammatory and antioxidant effects (Buckley and Libby, 2024). In addition, berberine–an isoquinoline alkaloid from *Coptis chinensis* and *Berberis* species–has garnered attention for its lipid-lowering and anti-inflammatory properties. Clinical trials and meta-analyses indicate that berberine significantly reduces total cholesterol, LDL, and triglycerides (while raising HDL) in patients with dyslipidemia, an effect partly due to upregulation of LDL receptor expression in the liver and modulation of AMPK-dependent lipid metabolism. In the arterial wall, berberine also acts as an antioxidant and NF-κB inhibitor, resulting in decreased oxidative modification of LDL and suppression of cytokine-driven vascular inflammation. These actions have been correlated with reduced plaque size and improved plaque stability in preclinical models (Figure 2) (Li et al., 2009; Rui et al., 2021; Ma et al., 2022). Other alkaloids, such as nuciferine (from lotus, *Nelumbo nucifera*), likewise inhibit inflammatory signaling and oxidative stress in vascular cells, contributing to anti-atherosclerotic effects (Xiao et al., 2023).

Empirically, diets or herbal therapies rich in these TCM phytochemicals are associated with lower rates of coronary artery disease (Moss and Ramji, 2016; Penson and Banach, 2021; Centers for Disease Control and Prevention, 2024). As research advances, specific natural metabolites are emerging as promising adjuncts or alternatives for atherosclerosis management alongside standard therapies. However, their exact clinical impact will need confirmation in large-scale trials.

### 2.3 Hypertension and endothelial dysfunction

Hypertension, or chronically elevated blood pressure, results from a combination of increased vascular resistance (often due to arterial constriction or stiffness) and sometimes elevated cardiac output. Endothelial dysfunction is a hallmark of hypertension – the impaired ability of blood vessels to dilate, usually due to reduced NO availability and a pro-inflammatory endothelium. TCM approaches have long included herbal therapies for “calming the liver” and improving circulation in hypertensive patients. Modern pharmacology is beginning to validate some of these botanical treatments.

Flavonoids have demonstrated notable antihypertensive effects attributable to their vasodilatory and antioxidant properties. Many flavonoids stimulate the endothelial production of NO by upregulating eNOS or by enhancing NO release, leading to vasorelaxation. For example, *in vitro* experiments show that quercetin and its glycosides cause endothelium-dependent relaxation via the PI3K/Akt-eNOS pathway (Das et al., 2023; Harahap et al., 2024; Xin et al., 2024). In hypertensive rat models, chronic administration of quercetin, puerarin, or rutin leads to lowered blood pressure, associated with improved endothelial function and decreased oxidative stress markers (Elbarbry et al., 2020; Chen et al., 2021). These effects are comparable to standard antihypertensives in some studies, though achieved through different mechanisms (e.g., antioxidant and anti-inflammatory pathways rather than direct effects on angiotensin or calcium channels) (Zheng et al., 2024; Jomova et al., 2025). Alkaloids like tetrandrine (from *Stephania tetrandra*) have calcium-channel blocking activity and have been used as antihypertensive agents in China. Berberine also shows benefit in hypertension; beyond its lipid effects, berberine improves endothelial function and induces vasodilation via increased NO and via activating AMPK (Amssayef and Eddouks, 2023). *In vivo* studies indicate that berberine can lower blood pressure in spontaneously hypertensive rats, with effects on the renin-angiotensin system and improved arterial compliance. Similarly, Morus alba leaf extracts (rich in alkaloid DNJ and flavonoids) have demonstrated blood pressure-lowering and endothelial-protective effects in animal models (Cao et al., 2021; Suadoni and Atherton, 2021; Wang Z. et al., 2024). Saponins also contribute to blood pressure reduction. Ginsenosides from Panax ginseng and Panax notoginseng have been found to promote vasodilation by increasing NO bioavailability and endothelial responsiveness. In hypertensive rodent models, ginsenoside Rg1 and Rb1 enhanced endothelium-dependent relaxation and reduced blood pressure, linked to activation of the Akt/eNOS pathway and inhibition of NADPH oxidase-derived ROS. Additionally, notoginsenoside R1 has been reported to improve microvascular endothelial function in animal models of hypertension, partly by reducing inflammation (e.g., lowering VCAM-1 expression as noted earlier). Astragalosides (from Astragalus membranaceus) and glycyrrhizin (from Glycyrrhiza uralensis) have shown vasoprotective effects as well, by ameliorating endothelial dysfunction and oxidative damage (Cao et al., 2021; Chu et al., 2023; Huang et al., 2023; Huang et al., 2024; Wang Y. et al., 2024). Some saponin-rich botanical formulations have historically been used to treat hypertension in TCM, and pharmacological studies now validate their efficacy in modern terms.

Collectively, diverse metabolites isolated from traditional Chinese medicine (TCM) exert a profound beneficial impact on the vasculature, characterized by enhanced endothelial function and diminished peripheral resistance. Their therapeutic action is predicated on a confluence of mechanisms, including increased nitric oxide (NO) bioavailability, reduced oxidative stress, and the intricate modulation of vasoactive signaling cascades. Consequently, these compounds exhibit significant antihypertensive properties. Given their potential to target the multifaceted pathophysiology of hypertension, either as monotherapies or as adjuncts to conventional treatments, they represent a promising therapeutic strategy. However, translating this substantial preclinical rationale into clinical practice necessitates rigorous, large-scale randomized controlled trials to substantiate their efficacy and safety in human populations.

### 2.4 Myocardial infarction and heart failure

Myocardial infarction (MI) and heart failure (HF) are closely intertwined clinical syndromes. An MI occurs when a coronary artery plaque ruptures or occludes the vessel, causing ischemic death of heart muscle. This often leads to adverse remodeling of the myocardium and can progress to HF. Heart failure, especially with reduced ejection fraction (HFrEF), is characterized by impaired pump function usually following myocardial injury, accompanied by neurohormonal activation and pathological remodeling (fibrosis, hypertrophy). TCM herbal therapies have been used traditionally to promote circulation, resolve blood stasis, and tonify the heart in these conditions. Modern investigations are revealing mechanistic support for some of these approaches in MI/HF models:

Preventing ischemia-reperfusion injury: Several TCM metabolites attenuate myocardial ischemia-reperfusion (I/R) injury – a key component of MI damage – by reducing oxidative stress and cell death. For example, luteolin (a flavonoid from Chrysanthemum and others) decreased infarct size and improved cardiac function in rat I/R models via Nrf2 activation and HO-1 upregulation (Baiyun et al., 2018; Oyagbemi et al., 2018). Salvianolic acid B (from Salvia miltiorrhiza) protects cardiomyocytes during I/R by scavenging ROS and inhibiting apoptosis through PI3K/Akt and ERK pathways (Dawuti et al., 2023). Notoginsenoside R1 also showed cardioprotective effects against I/R injury by modulating inflammation and apoptosis-related signaling (Chen Q. et al., 2024). These compounds essentially help the heart muscle better withstand the oxidative and inflammatory onslaught during reperfusion.

Limiting infarct expansion and fibrosis: After an MI, the reduction of infarct expansion and scar formation is crucial. Tanshinone IIA (a diterpenoid from Salvia miltiorrhiza) has been shown to limit infarct size and fibrosis in rodent MI models by anti-apoptotic and anti-fibrotic mechanisms (Wang N. et al., 2020). It can inhibit cardiac fibroblast activation via TGF-β/Smad pathways. Ferulic acid (from Angelica sinensis and Ligusticum chuanxiong) and astragaloside IV (from Astragalus) have similarly demonstrated reduction in cardiac fibrosis and improved post-MI ventricular function, linked to modulation of inflammatory cytokines and collagen synthesis (Lu et al., 2015; Liao et al., 2021).

Enhancing cardiac repair and function: Some TCM compounds may promote angiogenesis or myocardial repair. Panax notoginseng saponins have been reported to increase capillary density in ischemic myocardium and improve left ventricular function post-MI, potentially via upregulating VEGF and related pathways (Zhu et al., 2021). Erythropoietin-producing hepatocyte (Eph) receptor signaling modulators like icaritin (from Epimedium) showed cardioprotective effects by promoting endothelial repair after MI (Zhang et al., 2015).

In heart failure models, TCM-derived metabolites often exhibit anti-remodeling effects: reducing hypertrophy, fibrosis, and improving calcium handling in cardiomyocytes. For example, berberine was found to inhibit cardiac fibroblast proliferation and myofibroblast differentiation by modulating AMPK/PGC-1α signaling, helping to alleviate cardiac fibrosis in models of ischemic or diabetic cardiomyopathy (Hu et al., 2025). Corynoline (from *Corydalis* species) can increase the interaction between PPAR-α and NF-κB p65, thereby inhibiting NF-κB pro-inflammatory signaling and ameliorating cardiac inflammation (Wang M. et al., 2022). Ginsenosides such as Rg1 and Rd activate Nrf2-driven antioxidant responses in cardiac tissue, leading to higher levels of HO-1 and other antioxidants (Yao et al., 2022). Some saponins also modulate calcium handling and have anti-apoptotic effects in cardiomyocytes (through PI3K/Akt and other pathways) (Ghafouri-Fard et al., 2022). Salvianolic acids from *Danshen* (Salvia) can inhibit NF-κB and p38 MAPK signaling in cardiac fibroblasts, reducing their activation and collagen secretion (Dawuti et al., 2023). These multi-target effects (antioxidant, anti-inflammatory, anti-fibrotic, pro-angiogenic) converge to preserve cardiac structure and function, thereby offering therapeutic benefit in HF models.

In summary, across MI and HF, TCM metabolites work to limit the initial injury (e.g., reducing I/R damage), mitigate adverse remodeling (fibrosis, hypertrophy), and enhance residual function (through improved calcium dynamics and cardiometabolic effects). While these findings in cells and animal models are encouraging, translation to human patients will depend on well-designed clinical trials for post-MI or HF interventions using such compounds.

## 3 Clinical trials

Although much of the evidence for TCM natural metabolites in CVDs comes from preclinical research, a growing number of clinical studies and trials have begun to validate their efficacy and safety in humans. This section highlights key examples of TCM-derived metabolites that have made significant strides toward clinical translation, including berberine, quercetin, and the polyherbal Qili Qiangxin capsule, focusing on trial data, outcomes, and safety profiles (Bellavite et al., 2023).

### 3.1 Clinical evidence and translational successes

As a single-metabolite supplement, berberine has been used in East Asia for metabolic and cardiovascular health for decades, and Western interest has increased in recent years (Song et al., 2020). Multiple randomized controlled trials (RCTs) have evaluated berberine in conditions like dyslipidemia, type 2 diabetes, and heart failure. Some systematic reviews and meta-analyses have found that berberine significantly improves lipid profiles, with a decrease in total cholesterol, LDL, and triglycerides, while slightly increasing HDL. Notably, these improvements were not accompanied by serious adverse reactions; meta-analyses report no significant difference in overall incidence of adverse events between berberine and placebo, and no serious toxic reactions occurred (Ju et al., 2018; Ye et al., 2021; Blais et al., 2023). This suggests that berberine is generally well-tolerated at the doses used. Clinically, the lipid-lowering effect of berberine is roughly comparable to a low-to-moderate dose statin, but via a different mechanism–berberine upregulates hepatic LDL receptor expression and inhibits PCSK9, rather than inhibiting cholesterol synthesis. In addition, berberine has shown clinical benefits in improving metabolic diseases such as type 2 diabetes and non-alcoholic fatty liver disease, which are often comorbid with CVD. These multi-target effects may provide better cardiovascular risk reduction in patients with metabolic syndrome (Ilyas et al., 2020; Zhang et al., 2020; Zhao et al., 2021; Hu et al., 2022; Zhang et al., 2022; Nie et al., 2024). Overall, berberine stands out as a successful case of a natural metabolite with evidence from Phase II trials and meta-analyses supporting its efficacy (particularly in dyslipidemia) and an encouraging safety profile. It has not yet been incorporated into Western clinical guidelines, but in China berberine is sometimes recommended as an adjunct for dyslipidemia or diabetes management, especially when statins or metformin are insufficient or not tolerated.

Another promising compound is quercetin (a ubiquitous flavonoid, including in Ginkgo and Sophora). Early pilot trials of quercetin supplements in hypertensive patients showed modest reductions in blood pressure and improvements in endothelial function, without major side effects (Bondonno et al., 2016; Zheng et al., 2024). Quercetin’s clinical impact needs confirmation in larger trials, but it exemplifies how a well-known dietary phytochemical is being repurposed at higher doses for cardiovascular prevention.

Complex TCM formulas containing a variety of natural metabolites also play an important role in the treatment of CVDs. Qili Qiangxin (QLQX) capsules, which combine extracts of 11 herbs (e.g., *Panax ginseng*, *Astragalus membranaceus*, *Aconitum carmichaelii*, etc.), have been studied in the context of heart failure. Preclinical studies indicated that QLQX reduces myocardial fibrosis, inhibits cardiac hypertrophy, and improves ejection fraction in heart failure models. It acts on pathways like PI3K/Akt and AMPK (reducing inflammation and apoptosis) and enhances energetics in failing hearts (Fan et al., 2022; Wang T. et al., 2023; Wu et al., 2025). QLQX was recently evaluated in a large-scale Phase III clinical trial known as the QUEST study (Li et al., 2013). This multicenter, randomized, double-blind, placebo-controlled trial enrolled 3,110 patients with chronic heart failure with reduced ejection fraction (HFrEF) across 133 hospitals. The primary endpoint was a composite of rehospitalization for worsening heart failure or cardiovascular death. Clinical investigations reported in 2023 revealed compelling evidence for the efficacy of QLQX. When added to standard care, this botanical agent resulted in a significant 22% relative risk reduction for the primary composite endpoint of heart failure hospitalization or cardiovascular mortality compared to placebo (25.0% vs. 30.0%; hazard ratio [HR] = 0.78, 95% CI: 0.68–0.90; p < 0.001). Notably, this benefit encompassed a 24% reduction in heart failure hospitalizations and a 17% decrease in cardiovascular fatal events. Crucially, this therapeutic benefit was achieved with a favorable safety profile, as QLQX demonstrated tolerability comparable to placebo, with no significant hepatic or renal toxicity and no increase in arrhythmias or other major adverse events. By meeting stringent Western-style randomized controlled trial (RCT) standards and demonstrating efficacy on hard clinical endpoints, this multi-component traditional Chinese medicine (TCM) formulation challenges the conventional paradigm of single-molecule pharmacotherapy. The successful evaluation of QLQX not only paves the way for its incorporation into Chinese guidelines as an adjunct therapy for heart failure with reduced ejection fraction (HFrEF) but also establishes a robust methodological blueprint for assessing other complex TCM formulas in contemporary cardiovascular clinical trials. Table 3 lists the key clinical studies involving TCM metabolites, including the metabolite name, TCM source, study design, treatment duration, dosage, major outcomes (e.g., improved blood pressure, lipid profile), and any reported adverse effects.

**FIGURE 3:**
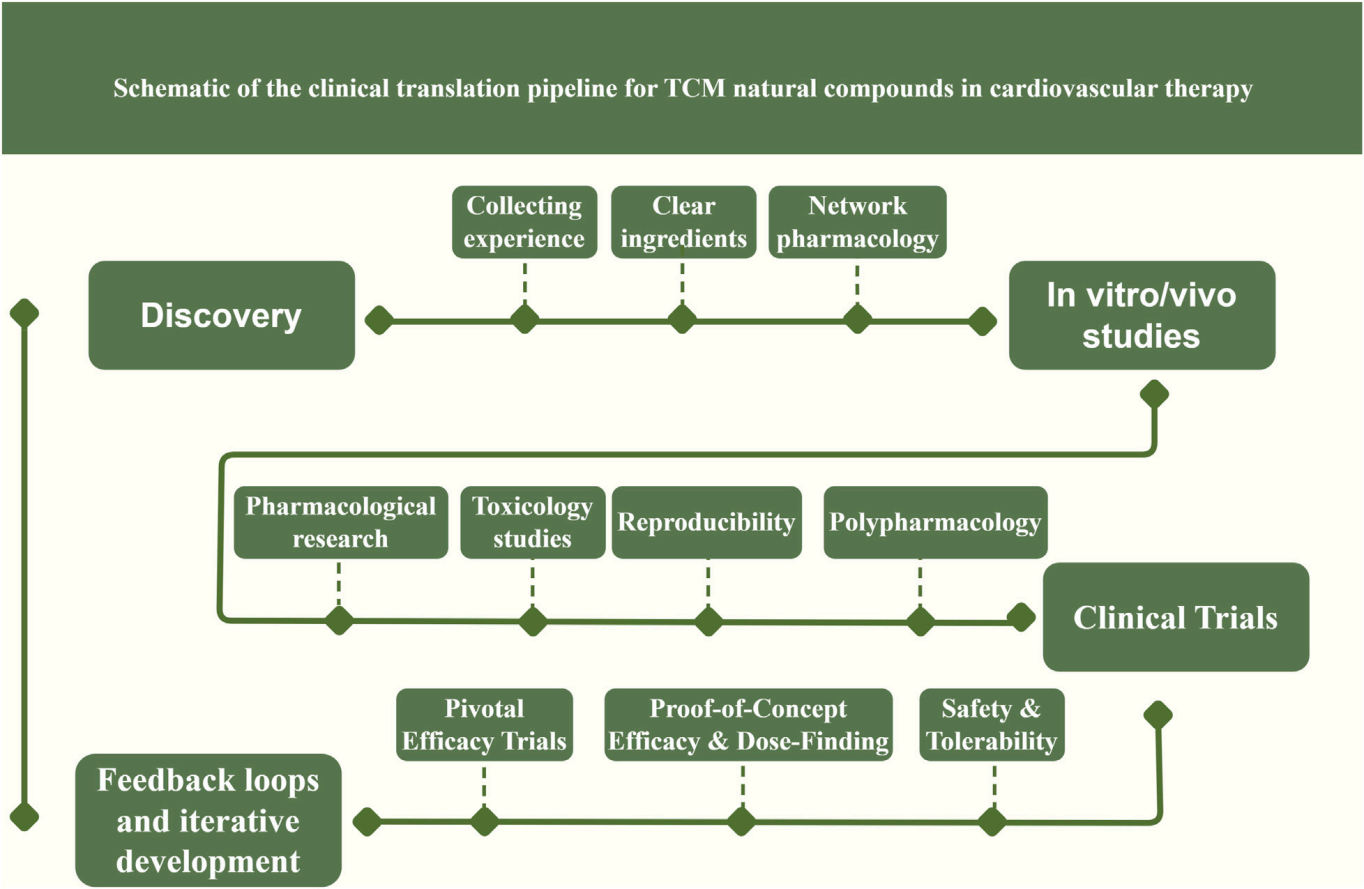
Schematic illustration of the clinical translation pipeline for TCM-derived natural compounds in cardiovascular therapy. Explanation: It outlines the sequential steps from initial compound discovery and characterization through mechanistic pharmacological studies, preclinical testing, formulation development, and phased clinical trials. These efforts ultimately culminate in regulatory approval of the new therapy and its integration into clinical practice guidelines. At the mechanistic study stage, emphasis is placed on target identification and pathway validation for key cardioprotective signaling networks (such as Nrf2, AMPK, NF-κB, and PI3K/Akt), utilizing systems biology approaches (omics and network pharmacology analyses) alongside *in vitro* assays and *in vivo* animal models to elucidate molecular mechanisms. Promising lead compounds then undergo rigorous preclinical efficacy and safety testing, concurrently with formulation development to enhance bioavailability and optimize pharmacokinetics. The pipeline’s clinical evaluation phase encompasses a series of human trials (Phase I, II, and III) to progressively establish safety and efficacy. Phase I trials focus on initial human safety assessment, tolerability, and pharmacokinetics. Phase II studies refine the dosing regimen and evaluate preliminary efficacy, often using biomarkers as surrogate endpoints to guide therapeutic assessment. Phase III comprises large-scale, endpoint-driven randomized controlled trials designed to confirm definitive clinical efficacy and safety in broad patient populations. In addition, the translation pipeline is iterative: feedback loops are incorporated such that insights from clinical trials (e.g., unexpected biomarker findings or safety signals) can prompt refinement of underlying mechanisms or necessitate reformulation of the compound. This adaptive process helps ensure that the most effective and safe TCM-derived interventions ultimately advance to regulatory approval and guideline-directed cardiovascular therapy.

However, not all clinical studies of TCM metabolites have been positive – some trials have yielded neutral results, possibly due to suboptimal dosing, bioavailability issues, or patient population factors. Therefore, continued rigorous research is needed. Overall, the trajectory is clear: TCM natural metabolites are beginning to transition from bench to bedside, supported by growing clinical evidence. The translation pipeline (Figure 3) typically progresses from identifying a promising metabolite or herbal formula, demonstrating efficacy in cellular and animal models, ensuring quality control in formulation, conducting early-phase human safety studies, then larger efficacy trials, and finally seeking guideline inclusion if successful.

### 3.2 Clinical application practice

TCM-derived compounds offer promising adjuncts in cardiovascular care. This section outlines how clinicians can apply these findings in practice – including practical dosing, integration with standard therapies, and safety monitoring – and suggests future research directions.

#### 3.2.1 Berberine clinical application and dosing

In clinical trials, berberine typically is administered at 500 mg two to three times daily (1–1.5 g total per day). For example, obese patients given 500 mg thrice daily for 12 weeks achieved significant improvements: ∼5 lbs weight loss and a 23% reduction in triglycerides with a 12% drop in total cholesterol. These metabolic effects approach the magnitude of first-line therapies – one trial even noted ∼18 mg/dL LDL reduction on average–suggesting berberine can complement conventional agents (e.g. adding to statins or metformin) for additional benefit. Indeed, combination studies report that berberine on top of statin therapy further improves lipid profiles compared to statins alone (Xiong et al., 2020; Zamani et al., 2022; Ge et al., 2024; Kong et al., 2025). Clinicians might consider berberine as an adjunct in patients with metabolic syndrome or hyperlipidemia who require extra risk factor reduction or cannot tolerate higher doses of standard drugs. Importantly, berberine should not replace evidence-based medications like statins but rather be integrated alongside lifestyle modifications and guideline-directed therapies.

#### 3.2.2 Qili Qiangxin capsule: integration into heart failure therapy

QLQX is a patented Chinese herbal capsule approved in China for chronic heart failure. Clinically, QLQX is used in addition to standard heart failure therapy (beta-blockers, RAAS inhibitors, diuretics, etc.), not as a standalone treatment. QLQX can be a complementary therapy in HFrEF, potentially bridging gaps in symptom relief and disease modification when optimal conventional therapy is insufficient. QLQX is administered orally in capsule form. Each capsule contains 0.3 g of herbal extract. Heart failure studies have used about 1.5–2 g per day of QLQX, typically divided as 4–6 capsules daily. For instance, earlier clinical trials often gave 4–6 capsules total per day (e.g. 2 capsules three times daily) in mild-to-moderate heart failure. In the recent large HFrEF trial, a higher dosage was employed: four 0.3 g capsules three times daily (total 3.6 g/day) alongside standard therapy (Leung et al., 2021; Huang et al., 2022; Nie et al., 2024; Tao et al., 2024). This intensive regimen was well tolerated, though investigators allowed dose reduction to 2–3 capsules TID if adverse. In practice, clinicians should start with the evidence-backed dosing (e.g. 4 capsules twice daily or three times daily, per severity) and adjust based on patient tolerance. Notably, the Chinese pharmacopeia recommendation for QLQX is 4 capsules three times per day, which aligns with the high-dose trial protocol. Integrating QLQX with Conventional Therapy: Patients receiving QLQX must continue on standard heart failure medications. In all major trials, QLQX was an adjunct to guideline-directed medical therapy (GDMT). The add-on of QLQX appears to confer additional benefit (e.g. fewer HF hospitalizations) without impeding standard drugs’ actions. The result is a more holistic therapeutic strategy–treating both the “root” (underlying myocardial remodeling) and “branch” (symptoms like edema) in TCM parlance, while fully maintaining modern medical therapy.

#### 3.2.3 Other cardiovascular integrations

Beyond heart failure, TCM compounds are being explored in coronary artery disease and hypertension as adjuncts to standard care. Tongxinluo was given as 1.04 g (4 capsules) three times daily for 12 months alongside all guideline-recommended medications, and it produced no increase in adverse events (Yao et al., 2020; Yang Y. et al., 2023; Yang Z. et al., 2023; Ouyang et al., 2025). These outcomes highlight that integrating TCM formulations with conventional therapies can yield additive benefits in ischemic heart disease, potentially by protecting microcirculation and reducing reperfusion injury. The combined use of Chinese herbal medicines and conventional antiplatelet agents represents a promising therapeutic strategy for atherosclerotic cardiovascular diseases (ASCVDs), demonstrating significant clinical benefits. This integrated approach offers dual advantages: enhancing antiplatelet efficacy while simultaneously mitigating bleeding risks. Extensive research confirms that specific herbs, such as Salvia miltiorrhiza, exert a synergistic effect when co-administered with drugs like aspirin or clopidogrel, leading to amplified suppression of platelet aggregation and a reduced thrombotic risk. For instance, extracts from S. miltiorrhiza, rich in tanshinones and salvianolic acids, have been shown to potentiate the effects of these antiplatelet medications. Importantly, this combination also provides superior bleeding risk control compared to alternative anticoagulation regimens. A notable clinical study demonstrated that patients receiving Naoxintong capsules (1.6 g/day, three times daily) plus aspirin (100 mg/day) exhibited a significantly lower incidence of severe bleeding than those on an adjusted-dose warfarin regimen (0% vs. 7.9%; OR = 0.921, 95% CI: 0.862–0.984, p = 0.028), providing robust evidence for the treatment’s safety profile. Furthermore, certain herbal formulations can improve vascular endothelial function. Research revealed that pretreatment with Trichosanthis Fructus pellets (1 g/kg/day) and aspirin (0.01 g/kg/day) in rats not only inhibited platelet aggregation but also enhanced endothelial integrity. Mechanistically, this combination therapy modulates key mediators such as thromboxane A2 (TXA2) and prostacyclin (PGI2), including their downstream effector cyclooxygenase-2 (COX-2), thereby contributing to its antithrombotic properties. Collectively, these findings underscore the substantial clinical value and potential of integrating Chinese herbal medicine with antiplatelet therapy for the prevention and management of ASCVDs.

Clinicians can accommodate such integrative approaches cautiously, ensuring that patients continue their ACE inhibitors, calcium blockers, etc. while an herbal remedy is tried. Given the lack of definitive data, TCM for hypertension should be considered complementary and secondary – never a replacement for proven pharmacotherapy.

#### 3.2.4 Safety, monitoring, and drug interactions

When integrating TCM-derived treatments, clinicians must monitor safety parameters and be mindful of herb–drug interactions. Both berberine and QLQX have favorable safety profiles in trials, but real-world use requires vigilance:

Hepatic and Renal Function: Berberine has not been associated with liver enzyme elevations or hepatotoxicity in clinical studies. Short-term trials (up to 3 months) reported no significant changes in liver or kidney function tests compared to placebo (Ji et al., 2025). Despite this, experts recommend periodic liver function monitoring if berberine is used long-term, especially in patients with underlying liver disease. This is a precaution to catch any idiosyncratic reaction early. Similarly, QLQX capsules have not shown hepatic or renal toxicity in controlled trials (e.g., adverse event rates were comparable to placebo over 18 months). Still, routine lab monitoring (metabolic panel, renal function) is prudent in heart failure patients adding any new therapy (Xing et al., 2022; Cheang et al., 2024).

Electrolytes and Hemodynamics: Patients on QLQX plus diuretics should have electrolytes closely followed, particularly potassium. The herbal components in QLQX promote diuresis (e.g. Alismatis in the formula) and enhanced natriuresis, which can potentiate the effect of loop or thiazide diuretics. There is a risk of additive hypokalemia when QLQX is combined with aggressive diuretic therapy, so clinicians should check potassium levels and adjust diuretic dose if needed. In practice, co-administration of QLQX with furosemide or hydrochlorothiazide may warrant more frequent electrolyte monitoring during the initial weeks. Conversely, if a patient is on an ACE inhibitor or spironolactone (risking hyperkalemia), the mild diuretic effect of QLQX might actually counterbalance potassium somewhat–but this interaction is not well quantified, so monitoring is still essential. Blood pressure and heart rate should also be observed when starting QLQX. Its vasodilatory and positive inotropic effects could in theory influence hemodynamics. While trials did not find significant hypotension or arrhythmias attributable to QLQX, a patient on multiple vasoactive drugs should be watched for blood pressure changes or palpitations. Notably, aconite (an ingredient in QLQX) is a potent cardiotonic herb; in improper doses aconite can cause arrhythmias, though in QLQX it is present in processed form (Fuzi) at safe dosages. Clinicians should still be alert to any new arrhythmic symptoms in patients on QLQX, especially if also on digoxin or other inotropes. It may be wise to obtain a baseline and follow-up ECG in HF patients when initiating QLQX, particularly if they have a complex arrhythmic history.

#### 3.2.5 Drug–herb interactions

In the context of integrated management strategies for cardiovascular and metabolic disorders, Drug–Herb Interactions manifest unique synergistic regulatory mechanisms alongside substantial clinical benefits. For hypertension control, there is robust evidence from randomized controlled trials supporting the efficacy of traditional Chinese medicinal formulations, such as Yiqi Huaju formula and Songling Xuemaikang Capsules. These formulations can modulate blood pressure through distinct pathways. Particularly noteworthy is that Bushen Qinggan demonstrate superior systolic pressure reduction compared to conventional antihypertensive regimens. Moreover, CGSHY herbal mixture exhibits comparable antihypertensive potency to the angiotensin-converting enzyme inhibitor enalapril, while coadministration of Jiangya Capsules with the calcium channel blocker nimodipine markedly enhances therapeutic outcomes in elderly patients with isolated systolic hypertension. Turning to secondary prevention in coronary artery disease, Xuezhikang significantly reduces coronary event rates among hypertensive survivors of myocardial infarction. Shenshao tablets substantially improve quality of life metrics in patients with stable angina pectoris, whereas Quyu Xiaoban capsules decrease angina frequency by suppressing platelet activation in unstable angina cases. Collectively, these findings underscore how Drug–Herb Interactions operate through multi-targeted mechanisms to offer promising integrated medical approaches for cardiometabolic disease management. Such interactions represent a compelling frontier for developing personalized treatment paradigms that leverage both pharmacological precision and holistic therapeutic principles.

Perhaps the most critical consideration is interactions between TCM compounds and prescription drugs, while potential adverse effects or complexities must also be acknowledged. Berberine is a known inhibitor of P-glycoprotein and certain CYP450 enzymes, which can lead to elevated levels of concurrent medications. A prominent example is the calcineurin inhibitor cyclosporine – coadministration of berberine has been shown to markedly increase cyclosporine blood concentrations, risking toxicity. Similarly, berberine may increase serum levels (and effects) of drugs like tacrolimus, digoxin, diltiazem, or certain statins by reducing their metabolism or efflux (Abushammala, 2021; Adiwidjaja et al., 2022; Nyulas et al., 2024; Zakiev et al., 2024). Clinicians should review each patient’s medication list for any narrow-therapeutic-index drugs. If a patient on warfarin, for instance, starts berberine, extra INR checks are indicated, as berberine could potentiate warfarin’s effect (reports of enhanced anticoagulation have been noted anecdotally). Berberine also has additive hypoglycemic action; a patient on insulin or sulfonylureas might experience lower blood sugars once berberine is added, so glucose should be monitored and diabetes medications adjusted to avoid hypoglycemia. QLQX being a multi-herb combination, has a lower risk of specific cytochrome-mediated interactions (since it’s not a single high-concentration alkaloid), but it can still interact pharmacodynamically with cardiac drugs. Its digitalis-like component (periplocymarin from Periploca) could synergize with prescribed digoxin, potentially increasing inotropic effect–caution is advised to watch for digitalis toxicity signs if patients are on both. However, in the large QLQX trial, many patients were on digoxin and no increased toxicity was reported, suggesting it’s generally safe. Nonetheless, prudent practice is to use the lowest effective digoxin dose if combining with QLQX, and monitor serum digoxin levels when available. QLQX’s vasodilator herbs (e.g. danshen) could also augment antihypertensive drugs, so blood pressure should be monitored to avoid hypotension when combining, say, QLQX with high-dose ACE inhibitors (Wang et al., 2025). Overall, open communication with patients about herbal and supplement use is essential: clinicians should ask specifically about TCM products, as patients may not volunteer this information, and incorporate that into medication reconciliation to manage interactions.

#### 3.2.6 General tolerability

Both berberine and QLQX are generally well tolerated. The most common side effects of berberine are mild gastrointestinal issues – e.g. constipation, nausea, or diarrhea – occurring in a minority of patients. These often subside with continued use or dose adjustment (starting at 500 mg twice daily with food can improve GI tolerance, then uptitrating to three times daily) (Li et al., 2023). QLQX capsule’s side effect profile in trials was similar to placebo. Some patients report dry mouth or gastric discomfort with QLQX, which can be mitigated by taking it after meals. Because QLQX contains multiple ingredients, there is a small risk of allergic reactions; clinicians should discontinue it if any rash or hypersensitivity signs appear. Importantly, any TCM compound should be avoided in pregnancy unless specifically studied, as many herbal components (including berberine) can cross the placenta. Berberine is contraindicated in late pregnancy and neonates due to risk of kernicterus (it can displace bilirubin). While young women are less likely to need these therapies for cardiovascular issues, clinicians should ensure non-pregnant status or discuss contraception if prescribing berberine to women of childbearing potential.

In summary, with appropriate monitoring – periodic labs, tracking vitals, and checking for drug interactions – berberine and QLQX can be safely integrated into practice. The active involvement of clinicians in monitoring is critical to catch any adverse effects early. This includes scheduling follow-ups a few weeks after initiation to reassess blood pressure, volume status, metabolic panel, and therapeutic drug levels as needed. Educating patients to report any unusual symptoms (e.g. dizziness, palpitations, muscle weakness that could indicate hypokalemia, etc.) further enhances safety.

## 4 Challenges and future perspectives

Despite the encouraging advances outlined above, significant challenges remain before TCM-derived therapies can be fully integrated into mainstream cardiovascular medicine. In this section, we discuss key hurdles and future directions: standardization and quality control of natural products, mechanistic complexity and polypharmacology, clinical translation and integration with current therapies, and others.

### 4.1 Standardization and quality control issues

Natural metabolites derived from botanical drugs can have significant variability in content and potency. Factors such as plant species (and potential misidentifications or adulterants), growing conditions, harvest time, and post-harvest processing all influence the concentration of active ingredients. This lack of standardization makes it difficult to ensure consistent efficacy and safety. For instance, the content of ginsenosides in ginseng can vary widely between sources, and flavonoid levels in *Scutellaria* root depend on growth and drying conditions. Without rigorous quality control, one batch of an herbal extract might be far less potent (or conversely, contain more of a toxic constituent) than another.

Furthermore, each botanical drug typically contains multiple active components. Isolating the effect of one specific metabolite can be challenging when others are present, potentially synergizing or antagonizing each other. Reproducibility of research is a concern: if studies do not fully characterize the herbal extract used, results cannot be reliably compared or replicated. Advanced analytical techniques (such as LC-MS metabolite profiling, NMR spectroscopy, and chromatographic fingerprinting) are increasingly necessary to profile and guarantee the composition of herbal products. Regulatory agencies will need to establish clear standards for botanical drug preparations, similar to Active Pharmaceutical Ingredient (API) standards for single compounds, to facilitate wider acceptance.

An additional complication is the presence of pan-assay interference compounds (PAINS) in some plant extracts. PAINS are promiscuous molecules that can nonspecifically affect multiple assay readouts, leading to false-positive signals (Baell, 2016; Bisson et al., 2016; Baell and Nissink, 2018; Sun et al., 2021). For example, certain polyphenols or quinones can aggregate proteins or generate assay-reactive species, giving an illusion of activity in screening tests. We have to be cautious that some reported “active” natural compounds might actually be PAINS. This highlights the importance of using appropriate controls (such as counter-screens) and applying PAINS filters during drug discovery efforts to avoid being misled by artifacts. By excluding such interference compounds early, researchers can focus on truly promising metabolites.

To ensure reliability, taxonomic authentication of the source plant is essential (e.g., voucher specimens, DNA barcoding) as well as chemical standardization of the extract. For well-known TCM herbs, official pharmacopeias (e.g., Chinese Pharmacopoeia) often specify marker compounds and minimum content levels (see Supplementary Table S2 for examples). Adhering to these standards is critical. For instance, using *Salvia miltiorrhiza* extract standardized to a certain percentage of tanshinones and salvianolic acids helps ensure consistency across studies.

Encouragingly, efforts are underway to improve reporting and reproducibility. A consensus guideline known as ConPhyMP (Consensus on Phytochemical Characterization of Medicinal Plant extracts) has been proposed to standardize how researchers describe plant materials and extracts (Heinrich et al., 2022). We have applied these principles in our review, documenting each botanical drug’s scientific name, part used, traditional preparation, and key phytochemical markers (see Supplementary Table S1). Moreover, triple-fingerprinting approaches (combining, for example, TLC, HPLC, and UV-visible spectra) and multi-marker quantification are recommended to fully characterize extracts (Hamburger, 2019; Bārzdiņa et al., 2022; Helmick et al., 2023; Riaz et al., 2023). By following such guidelines and transparently reporting all aspects of the plant material and extraction process, future studies will be more reproducible and easier to evaluate. Adopting the ConPhyMP checklist in peer review (as in this journal) is a positive step toward that goal.

Another critical challenge is the inherently poor bioavailability of many botanical metabolites, which limits their therapeutic consistency. Natural compounds often have low aqueous solubility or are rapidly metabolized, leading to minimal absorption *in vivo*. To address this, researchers are developing advanced delivery systems and chemical modifications. Nanoformulations – such as encapsulating phytochemicals into liposomes, nanoemulsions, or polymeric nanoparticles – can dramatically enhance dissolution, protect labile compounds, and improve membrane permeability. For example, the major Salvia miltiorrhiza diterpenoid tanshinone IIA has very low water solubility and oral absorption; it has been formulated into lipid-based nanocapsules, micelles, and solid lipid nanoparticles to boost its bioavailability (Zhang et al., 2013; Jia et al., 2024; Zou et al., 2024). Another strategy is to create more soluble derivatives or prodrugs. Converting tanshinone IIA into its sodium sulfonate form (sodium tanshinone IIA sulfonate, STS) greatly increases polarity and bioavailability, and indeed STS is used interchangeably with tanshinone IIA in studies and has been approved in China as an injectable drug (Shao et al., 2024; Xie et al., 2025). As a general proof of concept, the alkaloid berberine (another plant-derived drug with <5% oral absorption due to P-gp efflux) showed markedly enhanced uptake when delivered via liposomal nanoparticles (Shan et al., 2013; Zhang et al., 2019; Murakami et al., 2023a, b; Cui et al., 2024). By employing such nano-delivery systems or medicinal chemistry optimizations, researchers can significantly improve the pharmacokinetic profiles of herbal metabolites, ensuring more consistent potency and therapeutic levels *in vivo*.

### 4.2 Mechanistic complexity and polypharmacology

One challenge lies in the very strength of TCM metabolites: their polypharmacology (ability to hit multiple targets). From a scientific standpoint, it can be difficult to pinpoint the primary mechanism of action when an herb or metabolite affects numerous pathways simultaneously. Standard drug development often seeks a single target for a single disease pathway, but these natural agents defy that model. They may exert moderate effects on several pathways rather than a blockbuster effect on one. How do we rigorously demonstrate causality for each effect?

Polypharmacology (multi-target activity) is another double-edged aspect of botanical drugs that demands careful strategy in drug development. On one hand, having multiple targets can produce synergistic efficacy in complex diseases; on the other hand, it complicates pinpointing mechanisms and predicting off-target effects.

Network pharmacology and “omics” approaches are emerging as powerful tools to tackle this complexity. By using transcriptomic or proteomic profiling, researchers can see broad changes induced by a TCM extract, generating a signature of its action. Computational network models can predict which pathways are most crucial. Still, dissecting multi-component mixtures remains arduous. Instead of the old “one drug, one target” paradigm, network pharmacology operates on a “multiple targets, multiple effects” model–essentially replacing the single “magic bullet” with a coordinated “magic shotgun” strategy. In practice, this means computational and experimental methods are combined to identify the network of protein targets and pathways influenced by a given herb or its constituents. High-throughput screening (HTS) and phenotypic assays can be applied to profile natural compounds across many potential targets or cellular pathways, generating data that are then integrated into drug–target networks. This allows researchers to pinpoint which targets are critical for the desired therapeutic effect and which interactions might cause side effects. For instance, network-based analyses of a Danshen (Salvia) multi-component formula identified nine key compounds collectively modulating 42 cardiovascular-related genes, highlighting how a mixture’s efficacy arises from multi-target synergy (Li et al., 2011). Such insights guide rational development: one can prioritize compounds that hit multiple synergistic targets or design combination therapies that harness beneficial polypharmacology while minimizing promiscuous interference. By employing network pharmacology modeling and broad counter-screening early on, developers can manage polypharmacology—leveraging it to enhance efficacy (through synergistic target engagement) and using informatics to avoid trap compounds that bind irrelevant or harmful targets. This integrative strategy improves the chances that a candidate natural drug will have reproducible, mechanism-based effects even with multiple molecular targets, thereby facilitating its progression through the drug development pipeline.

Another issue is that polypharmacology can lead to off-target effects and interactions. While hitting multiple targets can be beneficial (tackling a disease from different angles), it also increases the risk of affecting an unintended pathway, possibly causing side effects. For example, a compound that is antioxidant and anti-inflammatory might also weakly interact with a hormone receptor or ion channel, with unknown implications.

From a research design perspective, carefully deconvoluting the contributions of each component in a herbal formula is valuable. Strategies include testing isolated constituents versus the whole extract, using knockdown of specific pathways in cells or gene knockout models in animals to see if the effect persists, and applying pharmacological inhibitors to block suspected mechanisms. Such approaches have started to be employed (e.g., using Nrf2-knockout mice to confirm a compound’s effects are via Nrf2).

Metabolomics has emerged as a promising approach for improving cardiovascular risk stratification, particularly in intermediate-risk asymptomatic populations. Recent studies employing high-performance analytical techniques such as mass spectrometry (MS) and nuclear magnetic resonance (NMR) spectroscopy have identified discriminative metabolites including sphingomyelins, lysophosphatidylcholines, and ceramides that correlate with coronary artery disease (CAD) progression. Another team applied ultra-high-performance liquid chromatography quadrupole time-of-flight mass spectrometry (UPLC-QTOF-MS) to analyze the metabolic components of traditional Chinese medicine (TCM) metabolites in rat biological samples (blood, urine, and feces), thereby exploring the mechanisms of natural metabolites' metabolism *in vivo* and their efficacy and effects (Tang et al., 2015b). The researchers also used ultra-performance liquid chromatography coupled with diode array detection and electrospray ionization mass spectrometry (UPLC-DAD-MS) to identify differences in natural metabolites derived from different plant sources (Tang et al., 2013; Yi et al., 2014). This innovative identification method has brought new opportunities for the study of natural metabolites. For instance, a novel risk prediction model based on plasma ceramide profiles was developed, demonstrating significant prognostic value for adverse cardiovascular events in cohorts validated across multiple centers. These metabolic biomarkers exhibit incremental predictive power when integrated with traditional risk assessment tools like the FRS and SCORE2 systems, as evidenced by net reclassification improvements exceeding 15% in meta-analyses involving over 10,000 participants. However, current implementation remains constrained by technical heterogeneity in sample preparation protocols and data interpretation standards. Future research should prioritize multi-center validation studies incorporating standardized metabolomic workflows while exploring synergies between Western biomarkers and therapeutic pathways that modulate lipid peroxidation and inflammation cascades.

In summary, while the multi-target nature of TCM therapies is promising for multi-factorial diseases like CVD, it complicates the mechanistic study. Embracing systems biology and network pharmacology will be crucial in fully understanding and optimizing these interventions.

### 4.3 Clinical translation and integration with western medicine

The ultimate challenge is translating promising natural metabolites into approved, widely adopted therapies. As the clinical evidence section highlighted, there are now successful examples (e.g., the QLQX heart failure trial, and growing data on berberine). But many hurdles remain on the road to regulatory approval and clinical guideline inclusion.

Firstly, robust clinical trials are needed for each candidate. This requires significant funding and logistical support, as well as overcoming any regulatory hesitations about herbal products. Many existing trials of TCM therapies have been small or of variable quality. Future studies must match the standards of pharmaceutical trials – large sample sizes, randomization, placebo-control, and clear endpoints.

Another consideration is botanical drug–drug interactions: many patients will use natural products alongside standard medications, so understanding interactions is critical. For instance, St. John’s Wort (a Western herb) famously induces drug-metabolizing enzymes and can reduce the efficacy of warfarin or statins. Similarly, compounds in grapefruit juice (furanocoumarins) inhibit CYP3A4 and raise levels of certain calcium blockers. For TCM herbs, there are examples like licorice (*Glycyrrhiza*) which can cause hypokalemia and affect digoxin toxicity, or ginseng potentially affecting coagulation. As TCM metabolites move into mainstream use, physicians will need clear information on any contraindications or interactions with conventional drugs.

Safety monitoring is also paramount. While many herbal medicines have been used for centuries, rare adverse effects might not be recognized without modern pharmacovigilance. For example, *Aconitum* (aconite) can cause severe arrhythmias if improperly processed – highlighting the need for quality control. Liver and kidney function should be monitored in trials, as there have been instances of herb-induced liver injury (usually idiosyncratic or due to adulterants). So far, most discussed compounds have shown good safety profiles in studies, but ongoing vigilance is required.

On the positive side, regulatory agencies in some countries are increasingly open to natural product drugs. For example, the Chinese FDA (NMPA) has approved certain TCM formula capsules for indication in heart disease (like QLQX). In the West, artemisinin (from *Artemisia annua*, discovered via TCM) has become globally recognized for malaria, and omega-3 fish oil (though not TCM, a natural product) is included in guidelines for post-MI patients. These cases show that if strong evidence is available, natural metabolites can gain broad acceptance.

Traditional Chinese medicine (TCM) has demonstrated unique advantages in the treatment of cardiovascular diseases, which have been reflected in numerous studies. In terms of antihypertensive effects, Bushen Qinggan granules have shown efficacy comparable to that of Western medicine and can significantly reduce systolic blood pressure, providing a new option for hypertension treatment. The mechanism of action may involve multiple targets and pathways, such as regulating vascular endothelial function and influencing the neuroendocrine system, thereby improving blood pressure regulation. In the treatment of heart failure, Kanlijian can increase left ventricular ejection fraction (LVEF), Shemnai injection can improve the average movement velocity of the mitral ring, and Qili qiangxin can reduce brain natriuretic peptide (BNP) levels, indicating its positive impact on cardiac function. This may be related to the overall concept of TCM in regulating the balance of yin and yang in the body, improving the circulation of Qi and Blood, and thus alleviating the burden on the heart, enhancing myocardial contractility, and ultimately improving symptoms of heart failure. For coronary heart disease treatment, Xuezhikang TCM can reduce coronary events and has shown good effects in multicenter studies involving a large number of patients. Its potential mechanisms of action may include antiplatelet aggregation, anti-inflammation, and lipid metabolism regulation, among others. These mechanisms help protect coronary artery endothelial cells, stabilize plaques, and reduce the risk of cardiovascular events.

Despite robust preclinical data for many TCM-derived metabolites, clinical validation remains limited or preliminary for a majority of these compounds. It is important to acknowledge this gap explicitly: numerous promising agents have not yet been tested in large, high-quality trials, or have only small studies with inconclusive results. For example, tanshinone IIA (from Salvia miltiorrhiza or Danshen) and various flavonoids (e.g. baicalin from Scutellaria or quercetin found in many plants) show cardioprotective effects in animal models, but lack robust randomized controlled trial (RCT) evidence in patients. Many such compounds are supported mainly by preclinical findings or small-scale clinical studies of variable quality. This underscores the need for large-scale, well-designed RCTs to confirm efficacy and safety. To address these gaps, future research should prioritize pharmaceutical-grade clinical trial design for TCM metabolites. Key considerations include: clear inclusion criteria (e.g. enrolling patients with defined cardiovascular conditions – such as chronic heart failure (NYHA II–III) or stable angina – who are already on standard therapy), sufficient treatment duration (e.g. at least 6–12 months of intervention, to capture meaningful clinical outcomes rather than short-term surrogate changes), and meaningful primary endpoints (such as improvement in exercise capacity or cardiac function, reduction in hospitalizations or major cardiovascular events, or other hard outcomes like all-cause mortality). For instance, a multicenter, placebo-controlled RCT could enroll several hundred heart failure patients on optimal therapy, randomizing them to add-on therapy with a purified TCM metabolite (at a standardized dose) versus placebo for 1 year. The primary endpoint might be a reduction in 1-year heart failure hospitalization rates or improvement in left ventricular ejection fraction, with secondary endpoints including quality-of-life scores and biomarkers. Similarly, another trial could test a flavonoid-rich extract in patients with coronary artery disease, with endpoints such as exercise tolerance (e.g. via treadmill test) and incidence of angina or acute coronary events. By designing large, rigorous trials with appropriate populations, durations, and endpoints, researchers can generate the high-level evidence needed for regulatory approval and inclusion of these natural metabolites in clinical guidelines. This approach will not only strengthen the credibility of TCM-derived therapies but also accelerate their integration into mainstream cardiovascular care, guiding future interdisciplinary collaboration in an evidence-based manner.

If we aim for global recognition of a TCM metabolite, it likely has to meet the strictest standards. This means well-characterized chemistry, demonstrated mechanism, clear clinical efficacy, and safety equivalent to current drugs. Developing a natural product into a pill with a defined dose that doctors anywhere can prescribe is a long but feasible process. Some companies and research consortia are now investing in “phytopharmaceutical” development – essentially taking promising herbal compounds through the full drug development pipeline (purification, formulation, trials).

In moving forward, integration between traditional medicine and modern biomedical science will be the key. Bridging the knowledge of experienced TCM practitioners (who can guide which herbs “work” in practice and optimal preparation methods) with cutting-edge research (target validation, medicinal chemistry optimization of natural structures, etc.) will accelerate progress. Although the concept of homology between medicine and food has brought great hope for the future of health and wellbeing, there are still many challenges to be met to fully release its potential. The first challenge is to carry out more rigorous scientific research to verify the safety and effectiveness of homologous substances in medicine and food; Relying on the rich resources of food-borne drugs and traditional Chinese medicine treasure house and using strict scientific verification, we can develop evidence-based and widely recognized new therapies.

Encouraging interdisciplinary collaboration is crucial: teams that include cardiologists, pharmacologists, botanists, and chemists have begun working together on these challenges. Such collaboration is leading to a new paradigm where natural multi-component interventions stand alongside single-target drugs in a complementary fashion. Physicians and patients are increasingly open to integrative approaches, especially as data supporting them become more robust. Ultimately, our goal is to improve cardiovascular health outcomes by whatever means prove effective.

## 5 Conclusion

Natural metabolites used in TCM show great promise as therapeutic agents in the fight against cardiovascular diseases. Over the past decades, substantial progress has been made in understanding their mechanisms and moving them toward clinical use. These metabolites, including flavonoids (e.g., quercetin, baicalin), saponins (e.g., ginsenosides, astragalosides), alkaloids (e.g., berberine, matrine), and others like polyphenols and terpenoids, exhibit a remarkable breadth of cardioprotective actions. They combat oxidative stress, quell inflammation, enhance endothelial function and nitric oxide bioavailability, modulate lipid metabolism to prevent atherogenesis, and attenuate fibrosis and adverse remodeling in the heart. Such a multi-target approach is particularly advantageous in complex cardiovascular disorders like atherosclerosis, myocardial infarction, and heart failure, where multiple pathological processes co-occur and drive disease progression. These compounds counteract fundamental pathological processes such as oxidative stress, inflammation, endothelial dysfunction, abnormal lipid metabolism, and cardiac remodeling. Recent evidence from preclinical studies and clinical trials underscores their therapeutic potential. For example, the alkaloid berberine improves lipid profiles and heart function, and the multi-herb formula Qili Qiangxin has demonstrated improved outcomes in heart failure patients. However, challenges such as poor bioavailability, complex multi-component formulations, and lack of standardization still hinder widespread use. Emerging strategies like advanced drug delivery systems and modern analytical techniques are being developed to overcome these obstacles.

In light of these findings, further development of cardiovascular therapeutics based on these natural metabolites is highly promising. One approach is to select these active molecules as lead compounds for new drug development, using medicinal chemistry to optimize their efficacy and pharmacokinetic properties. Another approach is to create multi-component formulations that incorporate several active compounds to achieve synergistic effects, akin to traditional herbal combinations. There are already examples of these strategies: for instance, ligustrazine (an active alkaloid from Ligusticum chuanxiong) has been developed into an injectable drug for ischemic stroke, and the Compound Danshen Dripping Pill (composed of Salvia miltiorrhiza and Panax notoginseng extracts) is an approved multi-component medicine for coronary heart disease in China. These cases demonstrate the feasibility of translating natural product compounds into effective single-agent or combination therapies. Therefore, continued efforts to explore chemical derivatives of these components and to design rational compound formulations could yield novel therapeutic options for cardiovascular disease in the future.

Importantly, an increasing body of evidence from *in vitro* experiments, animal models, and clinical studies provides support for the efficacy of these natural metabolites. These positive findings lend credence to the traditional uses of such remedies and provide a scientific rationale for their further development and integration into modern therapy. At the same time, reported safety profiles are generally favorable. With proper standardization and dosing, many natural metabolites appear to cause fewer severe side effects than some synthetic drugs (though vigilance is still required, as any bioactive substance can pose risks at high doses or in susceptible individuals).

The journey of bringing TCM metabolites into mainstream cardiovascular care is underway but not yet complete. Ongoing research is expected to expand the list of TCM-derived metabolites with evidence-based benefits, to optimize their formulations (for example, improving bioavailability of poorly absorbed molecules), and to clarify their roles in practice (whether as adjuncts to standard therapy to address residual risks like inflammation, or as alternatives in specific scenarios such as statin-intolerant patients). Continued high-quality clinical trials will be critical for garnering regulatory approvals and guideline recommendations. In moving forward, integration between traditional herbal medicine knowledge and modern clinical science will be key. By uniting the wisdom of TCM’s pharmacopoeia with rigorous scientific validation, we can enrich the therapeutic arsenal against CVD. Collaboration across disciplines and cultures is fostering a paradigm where multi-component natural interventions complement single-target drugs. Physicians and patients are increasingly receptive to these integrative approaches as the evidence grows more robust. Ultimately, the goal is to improve cardiovascular outcomes by all effective means, harnessing the best of both traditional and modern medicine.

In conclusion, the rich pharmacopoeia of traditional herbal medicine offers promising therapeutic candidates for cardiovascular disease, either as standalone agents or in synergy with conventional treatments. Bridging traditional knowledge with modern clinical science not only expands our therapeutic arsenal but also exemplifies a holistic approach to complex diseases that targets multiple pathways to achieve better patient outcomes. The advances reviewed herein provide a strong foundation and impetus for further exploration, ensuring that the future directions of cardiovascular therapeutics will include the wisdom of nature’s pharmacy, transformed by and validated through modern science.
